# Performance of a sensitive haemozoin‐based malaria diagnostic test validated for vivax malaria diagnosis in Brazilian Amazon

**DOI:** 10.1186/s12936-021-03688-0

**Published:** 2021-03-12

**Authors:** Gisely Cardoso de Melo, Rebeca Linhares Abreu Netto, Victor Irungu Mwangi, Yanka Evellyn Alves Rodrigues Salazar, Vanderson de Souza Sampaio, Wuelton Marcelo Monteiro, Fernando Fonseca de Almeida e Val, Anne Rocheleau, Priyaleela Thota, Marcus Vinícius Guimarães Lacerda

**Affiliations:** 1 Fundação de Medicina Tropical Dr Heitor Vieira Dourado, Manaus, Amazonas 69040-000 Brazil; 2 Universidade do Estado do Amazonas (UEA), Manaus, Amazonas 69040-000 Brazil; 3 Fundação de Vigilância em Saúde (FVS) - Manaus, Manaus, Amazonas 69093-018 Brazil; 4 Hemex Health, 4640 SW Macadam Avenue, Suite 250 , Portland, Oregon 97239 USA; 5 Instituto Leônidas & Maria Deane (ILMD) Fiocruz, Manaus, Amazonas 69057-070 Brazil

**Keywords:** Malaria, *Plasmodium vivax*, Diagnostic test, Haemozoin, Magnetic-optical detection

## Abstract

**Background:**

Vivax malaria diagnosis remains a challenge in malaria elimination, with current point of care rapid diagnostic tests (RDT) missing many clinically significant infections because of usually lower peripheral parasitaemia. Haemozoin-detecting assays have been suggested as an alternative to immunoassay platforms but to date have not reached successful field deployment. Haemozoin is a paramagnetic crystal by-product of haemoglobin digestion by malaria parasites and is present in the food vacuole of malaria parasite-infected erythrocytes. This study aimed to compare the diagnostic capability of a new haemozoin-detecting platform, the Gazelle™ device with optical microscopy, RDT and PCR in a vivax malaria-endemic region.

**Methods:**

A comparative, double-blind study evaluating symptomatic malaria patients seeking medical care was conducted at an infectious diseases reference hospital in the western Brazilian Amazon. Optical microscopy, PCR, RDT, and Gazelle™ were used to analyse blood samples. Sensitivity, specificity, positive predictive value (PPV), negative predictive value (NPV) and Kappa values were calculated.

**Results:**

Out of 300 patients, 24 test results were excluded from the final analysis due to protocol violation (6) and inconclusive and/or irretrievable results (18). Gazelle™ sensitivity was 96.1 % (91.3–98.3) and 72.1 % (65.0–78.3) when compared to optical microscopy and PCR, respectively whereas it was 83.9 % and 62.8 % for RDTs. The platform presented specificity of 100 % (97.4–100), and 99.0 % (94.8–99.9) when compared to optical microscopy, and PCR, respectively, which  was the same for RDTs. Its correct classification rate was 98.2 % when compared to optical microscopy and 82.3 % for PCR; the test’s accuracy when compared to optical microscopy was 98.1 % (96.4–99.7), when compared to RDT was 95.2 % (93.0–97.5), and when compared to PCR was 85.6 % (82.1–89.1). Kappa (95 % CI) values for Gazelle™ were 96.4 (93.2–99.5), 88.2 (82.6–93.8) and 65.3 (57.0–73.6) for optical microscopy, RDT and PCR, respectively.

**Conclusions:**

The Gazelle™ device was shown to have faster, easier, good sensitivity, specificity, and accuracy when compared to microscopy and was superior to RDT, demonstrating to be an alternative for vivax malaria screening particularly in areas where malaria is concomitant with other febrile infections (including dengue fever, zika, chikungunya, Chagas, yellow fever, babesiosis).

## Background

Malaria is the leading cause of death from parasitic infection worldwide [1]. Achieving control and elimination of malaria requires opportune and accurate determination of *Plasmodium* infection and, to an extent, differentiation of species, with the capacity for rapid screening becoming increasingly important for elimination. While malaria caused by *Plasmodium falciparum*, the principal cause of malaria mortality worldwide, is relatively well diagnosed in most areas using antigen-detecting rapid diagnostic tests (RDTs), other species are less well detected, particularly in remote settings [2]. *Plasmodium vivax*, the second most prevalent species of the six human malaria-causing *Plasmodium* species, is concentrated in Asia, Central and South America, the Western Pacific and the Horn of Africa [3] where it also imparts a significant health burden [4]. In 2019, 89.3 % of malaria cases reported in Brazil were of vivax malaria [5]. In many areas where both *P. falciparum* and *Plasmodium vivax* cause disease, malaria control efforts have effectively reduced the number of *P. falciparum* infections, but there has been an increase in the proportion of infections attributed to *P. vivax* [6].

Diagnosis, control and elimination of *P. vivax* is complicated by hard-to-detect low densities of circulating blood-stage parasites in many symptomatic patients and by its undetectable latent liver stage (hypnozoites) that results in relapses if not specifically targeted with 8-aminoquinolone drugs. Parasite densities of *P*. *vivax* are commonly much lower than *P*. *falciparum* in symptomatic infections [7], limiting the sensitivity of RDTs [8].

Because RDTs have poor sensitivity and molecular-based diagnosis are expensive for detecting some *Plasmodium* species [9], light microscopy remains the gold standard for malaria diagnosis in most parts of the malaria-endemic world [10]. However, its use depends on the availability of a well-functioning light microscope, electrical power, clean glass slides, immersion oil with appropriate optical properties, freshly filtered reagents for Giemsa staining, and importantly, a skilled microscopist. It is time-consuming and its accuracy diminishes in low parasitaemia [10].

Similarly, the performance of an RDT can be affected by multiple host and parasite factors [11]. Most RDTs rely on detection of parasite-derived protein histidine-rich protein 2 (PfHRP2), expressed only in *P*. *falciparum*, and the parasite’s metabolic enzymes, lactate dehydrogenase (pLDH) and aldolase, which are common to all species. HRP2-detecting tests are of limited utility in some regions because of their inability to detect *P. falciparum* parasites with *hrp2* and *hrp3* gene deletions [12]. The sensitivity of pLDH or aldolase-based RDTs for the detection of *P. vivax* infection is generally lower than that of PfHRP2-based RDTs for *P. falciparum* [8, 13]. Although polymerase chain reaction (PCR) is highly sensitive and specific for the detection and identification of malarial parasites [14], it requires sophisticated laboratory equipment and highly trained personnel [15]. For these reasons, the use of PCR is effectively limited to research settings and is not a viable option in the field. The loop-mediated isothermal amplification (LAMP) technique is the most commonly used isothermal strategy for malaria diagnosis. Commercial LAMP systems mainly rely on turbidity measurements, as these nucleic acid testing (NAT) reactions produce a large amount of DNA that reacts to produce magnesium pyrophosphate precipitate as a by-product [16, 17]. However, the existent commercially available LAMP-based malaria diagnostics are not yet fully portable, requiring laboratory instruments, high power use, specific reaction temperature settings, previous sample processing, and specialized training. At the moment, LAMP instruments are only bench-top apparatus and require miniaturization to fully meet the desirable qualities of an ideal commercial malaria diagnostic device [18]. Faster, easier and more sensitive diagnostics are indispensable for screening and diagnosis of *P. vivax*, for successful case management and in achieving malaria elimination [19].

Haemozoin is a paramagnetic crystal by-product of haemoglobin digestion by malaria parasites [20]. Because haemozoin is a highly specific biomarker for malaria infection, present in all *Plasmodium* species but in no other circulating pathogens, its detection is a promising approach to accurate diagnosis, especially in low parasitaemia. Attempts to develop portable, magneto-optical, point-of-care diagnostic technologies that detect haemozoin have been made but no commercial product has yet emerged. Earlier efforts to use haemozoin as a biomarker for malaria were largely unsuccessful [21, 22]. In addition, the parasite stage targeted influenced the results, such as immature forms that were established to have little or no detectable haemozoin [23]. Other methods lacked adequate specificity, minimum detection levels, had increased errors when detecting by resonance due to increased noise in the system, or were expensive [18, 20–22, 24]. The current device is vastly improved. It uses a very small volume of peripheral blood, has a lysis step that releases all the haemozoin into solution, and has powerful magnets that produce a magnetic field to align the haemozoin to inhibit the transmission of light through the solution. However, the size and morphology of haemozoin crystals may hold the key to identify accurately and differentiate species of malaria. Kumar et al.. observed that the sensitivity and specificity of Gazelle™ was 98 and 97 % compared to light microscopy, 82 and 99 % to PCR, and 78 and 99 % to RDT, respectively, in a predominantly *P. falciparum* endemic area [25].

Here, are reported the results from a study that assessed the accuracy of Gazelle™ (Hemex Health, USA), in detecting vivax malaria cases. The device is a late-stage portable prototype using magneto-optic technology for malaria diagnosis developed to detect haemozoin. The performance of Gazelle™ was compared with three current diagnostic methods: microscopy, RDTs and PCR.

## Methods

A comparative, double-blind study evaluating symptomatic individuals with suspected malaria, comparing optical microscopy, PCR, RDT, and Gazelle™ was carried out between June and October 2019 at *Fundação de Medicina Tropical Doutor Heitor Vieira Dourado* (FMT-HVD). The hospital is a reference centre for infectious disease care in Manaus, western Brazilian Amazon. Approximately 30 % of all cases of malaria in Manaus municipality are diagnosed at the FMT-HVD.

### Study subjects

Both male and female individuals aged ≥ 18 years seeking care at the FMT-HVD outpatient clinic were considered for enrolment. Per protocol, subjects were recruited, screened and enrolled by the investigator’s team at the study site, after the hospital’s routine malaria screening. All patients were informed of the study objectives and risks of participation. Participants were given adequate time to read the informed consent form (ICF) after which they were requested to sign it. Exclusion criteria were severe malaria, pregnancy and use of anti-malarials in the 30 days prior to study enrolment.

### Sample size

The sample size was calculated based on the assumption of 95 % confidence interval, an estimated 10 % accuracy (95 % CI width ≤ 10 %) and allowed for up to 10 % of subjects to be excluded because of incomplete or missing data. This translated to a minimum of 100 positive and 200 negative cases of malaria.

### Sample analysis

On study admission, immediately before drug administration, trained laboratory technicians collected whole blood samples in EDTA. Microscopy was the method used to confirm vivax malaria status of the participants before recruiting them into the study. Blinded study microscopists analysed all samples after slide preparation (thick and thin smears). Collected blood samples were stored at 4 °C and tested by RDT and Gazelle™ within utmost 4 h of collection. Aliquots of the whole blood samples were stored at − 20 °C until needed for DNA extraction for the subsequent molecular diagnosis of malaria by PCR analysis. All testers were blinded to the malaria status of the samples.

### Gazelle™

**Fig. 1:**
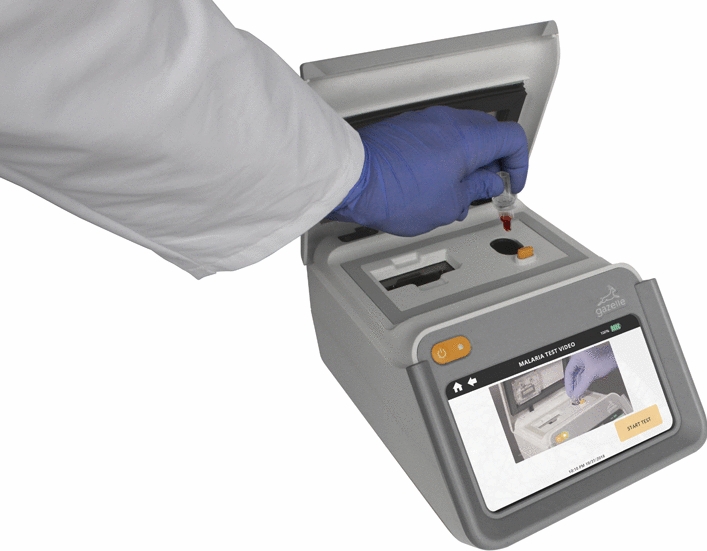
The Gazelle™ Device. Battery-powered equipment that can run 200 tests independently. The diagnostic reader can store or upload patient data to a phone or computer for later storage in the Cloud. GPS location, useful for epidemiological studies, can also be saved

**Fig. 2:**
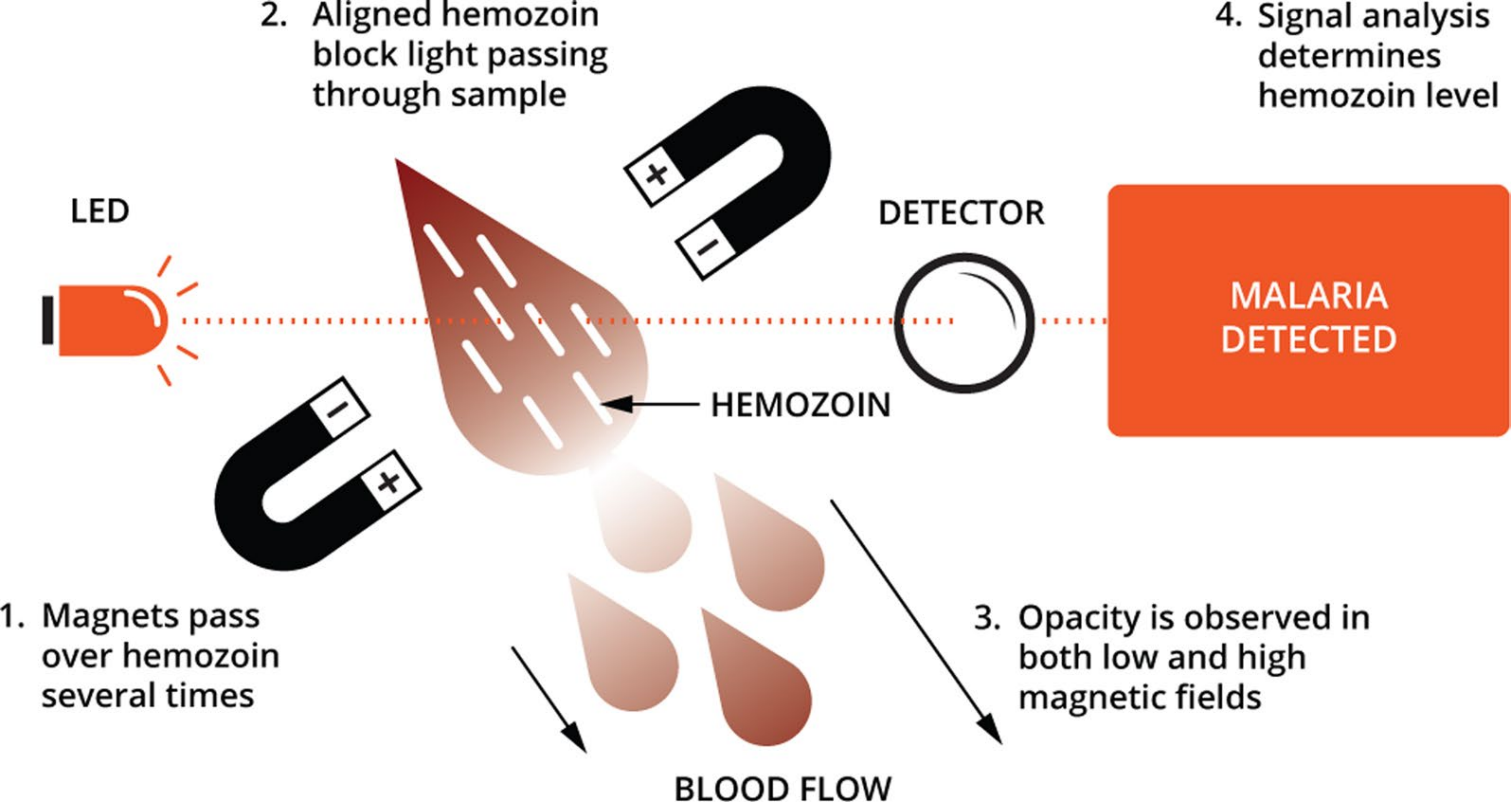
Principle of operation of Gazelle™. The device uses alternating magnetic fields to align haemozoin so that it blocks transmitted light. The light signal detected at the detector is inversely proportional to the amount of haemozoin present in the sample

The Gazelle™ technology is housed in a portable, point-of-care device (Fig. 1) and detects malaria infections based on the magneto-optical concept for the detection of paramagnetic haemozoin, a biomarker that appears very early during the life cycle of the malaria parasite. This being a prototype device, at the moment it cannot distinguish between *P. falciparum* and *P. vivax* haemozoin. When haemozoin, which is partly composed of iron, is subjected to an intense magnetic field the haemozoin crystals align in a manner that increases the opacity of the blood sample (Fig. 2). The opacity of the sample is measured in both a high magnetic field and a very low or no magnetic field using a LED and photodetector to determine the presence of haemozoin. The decrease of light reaching the photodetector in the presence of the high magnetic field is directly proportional to the amount of haemozoin present. If there is no change with variation in the magnetic field, then there is no haemozoin detected. An algorithm uses the optical information to determine the presence or absence of malaria.

**Fig. 3:**
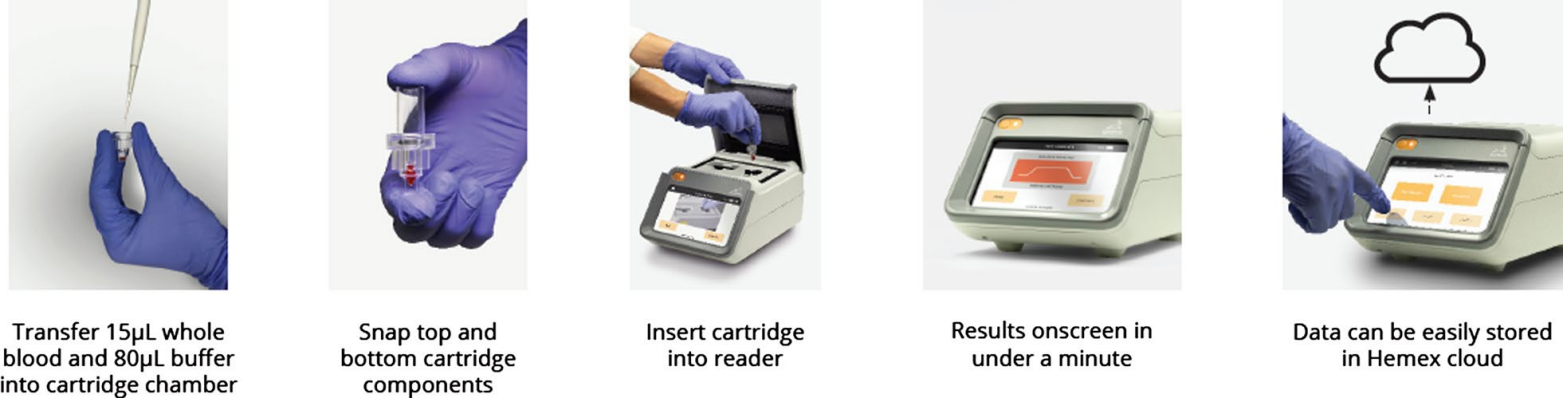
Steps involved in diagnosing malaria using the Gazelle™ device

For Gazelle™ testing, blood samples were prepared by pipetting 80 µL of malaria diluent (2 % Triton in water) and 15 µL of blood into a cuvette (Fig. 3). The cuvette containing the diluent/blood mixture is then loaded into the reader that automatically analyses the samples. Results appear on the Gazelle™ screen in about one minute and can either be stored in the reader (has internal storage), locally printed, or transmitted through Bluetooth or Wi-Fi connection. Another feature of this diagnostic device is that it can operate on either electric or lithium battery power, making it versatile for point-of-care use in remote locations with no electricity. This device can be recharged using a standard micro-USB charger similar to that used for android phones. Voltage drift threshold was set at 0.9 V for this study. The algorithm has limits to prevent the use of samples with too high voltage drift. The reader and disposables do not require special storage and can operate in temperatures of up to 45 °C [25].

### RDT

Venous blood samples were tested using the Malaria Ag Pf/Pan RDT (SD Bioline, Standard Diagnostics Inc., Gyeonggi-do, Republic of Korea) according to the manufacturer’s instructions. The RDT kits were stored at the recommended temperatures and were used before the expiration date.

### Microscopy

Thick blood smears were prepared, stained by Giemsa [26] and examined by two experienced microscopists, who counted the number of parasites per 200 or 500 leukocytes, depending on parasite density. Parasite density (parasites/µL) was determined by counting the number of parasites per leukocyte in high magnification fields with the assumption of 6,000 leukocytes/µL of blood [27]. In the event of a discrepancy > 10 % between the two, a third reading by an independently trained microscopist was performed using the Obare Method Calculator (version 1.0) to resolve the discrepancy [28].

### PCR

Extraction of total DNA from 200 µL of whole blood was performed using the QIAamp DNA Blood Mini Kit (Qiagen®, USA), according to the manufacturer’s protocol. QMAL Taqman qPCR was used to detect *Plasmodium* species by targeting a conserved region of the 18 S rRNA gene in the extracted DNA samples. The following probes and primers were used to analyse the extracted DNA samples: Fw-TTA GAT TGC TTC CTT CAG TRC CTT ATG; Rev-GT TGA GTC AAA TTA AGC CGC AA; FAM - TCA ATT CTT TTA ACT TTC TCG CTT GCG CGA - BHQ1 (wobble R = A/G) [29]. To quantify the 18 S rRNA gene copy numbers in each experiment, three dilutions of plasmids containing the respective targeted region were included in triplicates (10^2^, 10^4^, 10^6^ copies/µL). Briefly, 4 µL of DNA samples and plasmids were used in a total reaction volume of 12 µL to screen for malaria cases.

All qPCR assays were run in the 7500 Fast Real-Time PCR System (Applied Biosystems, CA, USA). The primer and probe sequences, the composition of reaction mixes, PCR profiles and the detection limit for each assay used were as described by Almeida et al. [7].

### STARD adherence

**Fig. 4:**
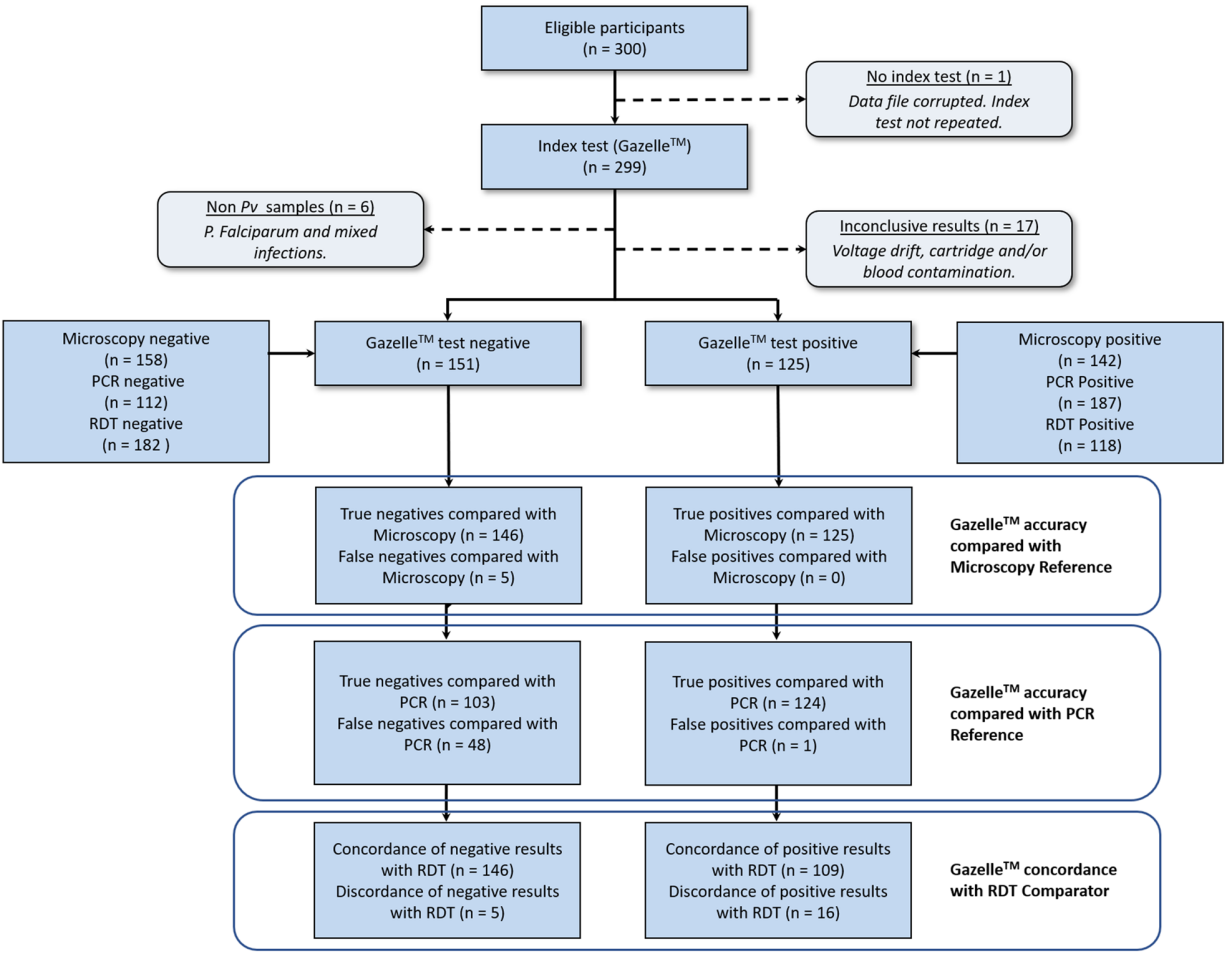
STARD Flowchart

STARD (Standards for Reporting of Diagnostic Accuracy Studies) shown in Fig. 4 details study test results, including an analysis of accuracy compared with reference standards for microscopy and PCR (true and false positives and negatives), and comparison of results with RDT as a non-reference comparator (concordance and discordance between Gazelle™ and RDT results).

### Ethical considerations

The Ethics Review Board of the *Fundação de Medicina Tropical Dr. Heitor Vieira Dourado* (FMT-HVD) approved the study (approval number 3.374.472/2019). Subjects were informed of the study objectives and gave their consent for participation through written and signed informed consent forms before study procedures. Subjects diagnosed with malaria were treated according to the Brazilian Ministry of Health guidelines [30].

### Analysis populations

Table 1 describes subject enrolment and defines three analysis populations. The full analysis population (FAP) includes all enrolled subjects tested. The per protocol population (PPP) includes all subjects with conclusive Gazelle™ test results. The *P. vivax* population (PVP) excludes all subjects with *P. falciparum* or mixed infections. The PVP is the primary population analysed in this study.

**Table 1:** Enrolment and analysis populations

Candidate subjects presenting for enrolment in the study	300
Total number of subjects enrolled and tested (full analysis population)	300
Subjects excluded from full analysis population	18
Reason: Data file not retrievable	1
Reason: Inconclusive Gazelle™ test result	17
Subject tested negative for malaria by other methods	12
Subject tested positive for *P. vivax* by other methods	3
Subject tested positive for *P. falciparum* by other methods	2
Number of subjects included in full data analysis (per protocol population)	282
Additional subjects excluded from the *P. vivax* data analysis subset	6
Reason: Subject tested positive for *P. falciparum*^a^	3
Reason: Subject tested positive for *P. falciparum* and *P. vivax*	3
Number of subjects included in*P. vivax*data analysis sub-set (*P. vivax*population)	276

^a^These 3 subjects infected with *P. falciparum* are in addition to the 2 subjects with *P. falciparum* excluded from the full analysis due to inconclusive Gazelle™ test results

### Statistical analysis

Gazelle™ diagnostic performance was measured by determining its sensitivity, specificity, likelihood ratios (LR), positive predictive value (PPV), negative predictive value (NPV), and detection accuracy. Gazelle™ was compared to RDT, to optical microscopy and PCR. Likelihood ratios for positive (LR+) and negative (LR−) test results were considered good when LR + was > 10, and LR− <0.1 [31]. Diagnostic accuracy was assessed through receiver-operating characteristics (ROC) curves and areas under the curve (AUC) interpreted as follows: 0.9-1.0, excellent; 0.8–0.9, very good; 0.7–0.8, good; 0.6–0.7, sufficient; 0.5–0.6, bad; <0.5, test not useful [32].

The two-graph ROC analysis enables the visualizing of sensitivity and specificity curves on a single graph, according to the range of values of the new test given the reference test. This provides a visualization for the specificity-sensitivity ‘optimal’’ trade-off in determining the best and acceptable cut-off for the new test to be used in practice against the already established gold standards available. The parasite detection parameters for the Gazelle™ test against microscopy and PCR were determined by a two-graph ROC analysis [32]. Results were considered statistically significant for *p* < 0.05. Analyses were performed on STATA (v13.1, StataCorp, College Station, TX, USA).

## Results

### Demographics

From the 300 patients that were tested, 24 samples were excluded from the final analysis: 1 had no results because of a corrupted data file, which was irretrievable; 17 due to inconclusive results (voltage drift, cartridge and/or blood contamination); 3 were infected with *P. falciparum*; and, 3 with mixed infections of *P. vivax* and *P. falciparum* (Table 1). Since this was a double-blind study, no repeat tests were performed on the 18 inconclusive and/or irretrievable test results that were excluded. The on-site study team were blind to the Gazelle™ test results when doing the tests.

Of the 276 subjects whose test results were analysed per protocol, 163 (59.1 %) were male. The study participants were aged between 18 and 79 years with a mean age of 40.4 (± 18.8) years. Out of the total number of subjects included in the *P. vivax* data analysis, 168 (60.9 %) reported one or more previous episodes of malaria prior to the current infection. The mean parasite density was 6,220 parasites/µL (± 8,254). Patient demographic and malaria infection characteristics are presented in Table 2.

**Table 2:** Demographic and malaria parasite characteristics – *Plasmodium vivax* population

Characteristic	Value, n (%)
Gender	
Male	163 (59.1)
Female	113 (40.9)
Age (mean, SD)	40.4 (± 18.8)
Previous malaria episode	
Yes	168 (60.9)
No	108 (39.1)
Parasite density per µL (mean, SD)	6,220 (± 8,254)
Parasite range (min, max)	18–73, 815
Parasitaemia density distribution (parasite/µL)	
< 1,000	34 (26.1)
1,000–10,000	90 (69.2)
> 10,000	6 (4.6)

*n* number of subjects,* min * minimum value,* max*  maximum value,* SD * standard deviation

### Malaria detection

Malaria detection characteristics such as mean parasite density, range and density distribution are shown in Table 2. Of the 276 subjects in the PVP, malaria was detected by RDT in 109 (39.5 %) subjects, by microscopy in 130 (47.1 %) subjects, by PCR in 172 (62.3 %) subjects, and by Gazelle™ in 125 (45.3 %) subjects.

### Microscopy as gold standard versus Gazelle™ and RDT

**Fig. 5:**
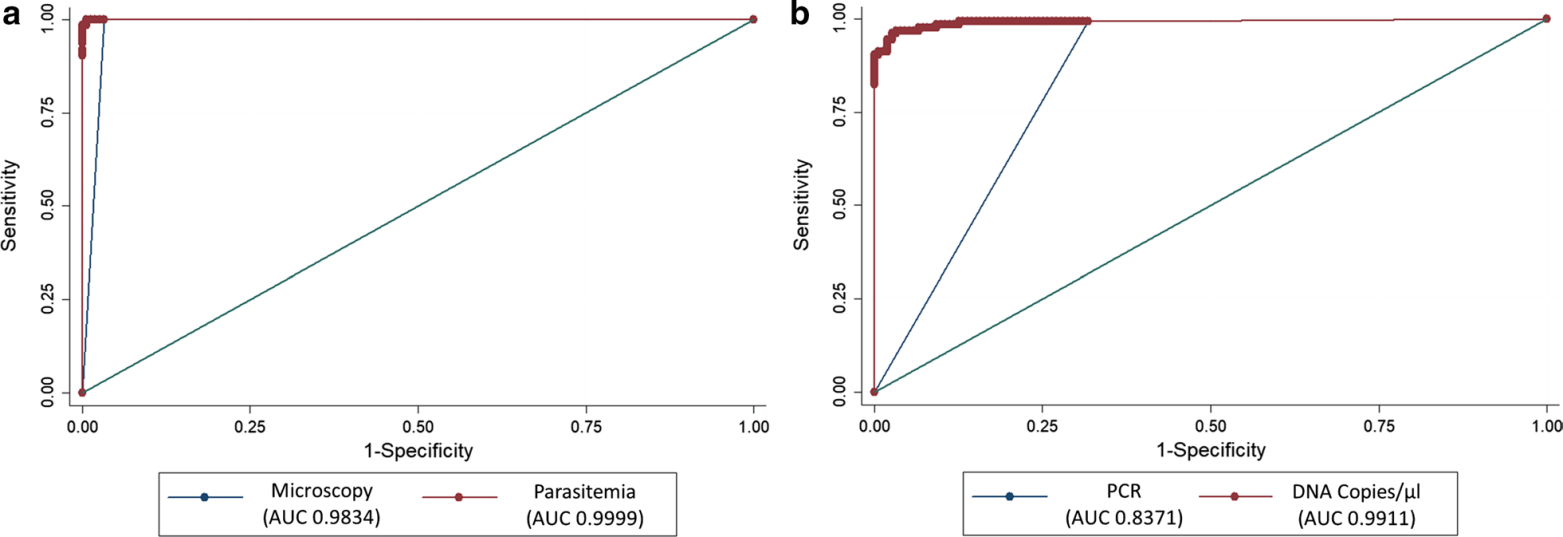
ROC analysis: Gazelle™ compared with microscopy and PCR. **a** With an AUC of 0.9834, Gazelle™ produced results that were comparable to microscopy. **b** With an AUC of 0.8371, Gazelle™ was less sensitive than PCR but as specific

When microscopy was positive for malaria, Gazelle™ produced a positive result for 96.2 % of the samples (125 of 130 tests). On the other hand, when microscopy was negative for malaria, the Gazelle™ result was 100 % consistent (146 of 146 tests). A ROC analysis of Gazelle™ against microscopy showed an accuracy of 98.3 % (Fig. 5a). In the five instances when the Gazelle™ result was negative and microscopy was positive, the microscopically observed parasite counts were low, ranging from 18 to 174 parasites/µL. For the tests where microscopy was positive and RDT was negative, the range of parasitaemia varied between 18 and 2,805 parasites/µL.

Sensitivity and specificity of Gazelle™ test results compared with optical microscopy were 96.2 and 100 %, whereas for RDTs they were 83.9 and 100 %, respectively (Table 3). PPV for Gazelle™ and RDTs, when compared to microscopy, were both 100 % whereas the NPV was 96.7 % for Gazelle™ and 87.4 % for RDTs, respectively. The overall accuracy of Gazelle™ was 98.2 % whereas it was 92.4 % for RDTs (Table 3).

**Table 3:** Performance of Gazelle™ compared to RDT with microscopy as gold standard

n = 276	Gazelle™	RDT
Sensitivity	96.2 % 125/130	83.9 % 109/130
Specificity	100 % 146/146	100 % 146/146
Positive predictive value	100 %	100 %
Negative predictive value	96.7 %	87.4 %
Likelihood ratio of a negative test	0.04	0.16
Accuracy (ROC analysis)	98.2 % 271/276	92.4 % 255/276

### PCR as gold standard versus Gazelle™, RDT and microscopy

When PCR was positive for malaria, Gazelle™ was positive 72.1 % of the time (124 of 172 tests). When PCR was negative for malaria, the Gazelle™ was negative 99.0 % of the time (103 of 104 tests). Further ROC analysis of Gazelle™ against PCR showed an AUC of 0.837, meaning that the Gazelle™ test was slightly less sensitive than PCR but very specific and with very good accuracy (Fig. 5b). Of the 48 tests positive by PCR and negative by Gazelle™, all were negative by RDT, and 5 were positive by microscopy.

Sensitivity and specificity of Gazelle™ test results compared with PCR were 72.1 and 99 %, whereas, for RDTs they were 62.8 and 99 %, and for microscopy, they were 75 and 99 %, respectively (Table 4). The overall accuracy of Gazelle™ was 82.3 % whereas it was 84.1 % for microscopy and 76.5 % for RDTs.

**Table 4:** Performance of Gazelle™ compared to microscopy and RDT with PCR as gold standard

n = 276	Gazelle™	Microscopy	RDT
Sensitivity	72.1 % 124/172	75.0 % 129/172	62.8 % 108/172
Specificity	99.0 % 103/104	99.0 % 103/104	99.0 % 103/104
Positive predictive value	99.2 %	99.2 %	99.1 %
Negative predictive value	68.2 %	70.6 %	61.7 %
Likelihood ratio of a positive test	75.0	78.0	65.3
Likelihood ratio of a negative test	0.28	0.25	0.38
Accuracy (ROC analysis)	82.3 %	84.1 %	76.5 %

For the sub-set of 48 tests where the PCR result was positive and Gazelle™ was negative, the average PCR count was 326.65 DNA copies/µL (or only 0.014 % of the average PCR count) with a median of 19.73 DNA copies/µL.

## Discussion

Accuracy is essential for rapid and timely diagnosis and treatment, identifying and interrupting malaria transmission in the community. Low parasite density infection continues to challenge diagnostic accuracy and obstruct malaria elimination efforts. Highly sensitive, rapid and affordable diagnostic tools are urgently needed to detect and improve the clinical management of *P. vivax* infections in endemic regions.

Gazelle™, a novel point-of-care, haemozoin-based malaria diagnostic device was evaluated, and compared to microscopy, RDT, and PCR. Sensitivity and specificity of Gazelle™ was nearly as sensitive and was as specific as expert microscopy and considerably more sensitive than RDTs, the most used diagnostic tools at community health centres. In addition, Gazelle™ had advantages in terms of speed, with a turn-around time of 90 s. Although relatively low cost, microscopy is rarely used in remote areas because infrastructure and skilled personnel are lacking, and electrical power supplies are often erratic or unreliable [33]. Furthermore, microscopy is labour-intensive, time-consuming and operator-dependent compared to RDTs [34, 35].

From this field trial study, the Gazelle™ outperformed RDTs, detecting *P. vivax* parasite densities down to 72 parasites/µL. These results affirmed the superiority of the test against widely used conventional diagnostic methods. Studies by Kumar et al. [25] demonstrated that the Gazelle™ test device detected as low as 50 cultured *P. falciparum* parasites/µL with 95 % accuracy; as low as 35 *P. vivax* parasites/µL from Indian patients could be detected with 100 % accuracy by the device. The difference in the limit of detection (LOD) between this study and the Indian study may be due to differences in parasite burden and parasite characteristics  of the *P. vivax* strains prevalent in the two regions. Dilution experiments elsewhere, applying the magneto-optical concept used in this device, suggest a LOD for Gazelle™ could go to as low as potentially < 20 parasites/µL with higher blood volume and trophozoite/schizont parasite stages [36]. These LODs, which correspond to approximately 20 pg/µL of haemozoin, are better than that of RDTs and compare favourably with the LOD achievable by expert microscopy [36, 37]. The cost of the test on the device is about $1 per test, and very much comparable to the cost of a single RDT and microscopy [25].

An attempt to determine the device cut-off limit relative to microscopy and PCR diagnostic techniques was also done. The parasitaemia and PCR copies raw data obtained from laboratory analysis ranged into thousands (parasitaemia) and millions (PCR copies). The identification of a precise cut-off using a cut-off graph plot was however based on too many points with little gain in accuracy. Subsequently, intervals of 500 were used, making 500,000 PCR copies/µL as the maximum value. With this modification, the “optimal cut-off” for Gazelle^TM^ test use was determined to be slightly lower than 100 parasites/µL and 1,000 copies/µL relative to microscopy and PCR, respectively. Further studies in different malaria transmission settings are necessary to validate the proposed “optimal cut-off” and LOD.

The current Gazelle™ prototype provides only qualitative malaria diagnosis without species differentiation, with detection of *P. falciparum* demonstrated elsewhere [25]. In its current form, it can be used in POC (point of care) to quickly screen for malaria infections from other tropical diseases that present with similar symptoms, including dengue, zika, chikungunya, Chagas, yellow fever, babesiosis. The successful incorporation of haemozoin’s magnetic properties and magneto-optics into a portable diagnostic device should help in the fast and accurate diagnosis of malaria cases in the community, while the capacity for species differentiation currently under development is necessary for management in areas with multiple endemic species. Future improved versions of similar Magnetic-optical detection (MOD) -based diagnostics systems are likely to be more specific, possibly using differing haemozoin crystal morphology to distinguish species [38]. Perhaps the size and morphology of haemozoin crystals may hold the key to accurately identifying and differentiating parasite developmental stages and species. The short time to result and higher sensitivity for *P. vivax* have potential to address the inadequacies of current RDTs in community-based vivax management and may prove useful in scenarios such as airports or borders in elimination settings where large numbers of people must be rapidly screened each day.

For operational purposes, the samples used for all diagnostic tests were venous blood. For malaria diagnosis in the field peripheral capillary blood is used, which could be a limitation of the study’s current sample collection and analysis design in that malaria parasite concentrations in capillary and venous blood differ. However, the diagnostic device can use small volumes of blood such as those obtained from finger prick (15 µL) for analysis. Despite all tests having been performed  with venous samples, future studies will need to address this issue by comparing the diagnostic potential of Gazelle™’s device  using blood from both collection sites. Expanding the capacity to diagnose and differentiate between species should increase  the Gazelle™ use as a POC testing device in field settings where mixed infections occur, or in areas where several species are in circulation. The results obtained in this study could be restricted to the malaria strains present in the Brazilian Amazon, and the application of this device in other countries should be addressed. Despite these limitations, results from this study support the use of Gazelle™ in areas with limited qualified human resources and electrical power supply, and thereby increase capacity of malaria diagnosis in areas where transmission mostly occurs.

## Conclusions

This study demonstrated that Gazelle™, the first commercially developed haemozoin-detecting instrument for the diagnosis of malaria, outperformed RDTs and was nearly as sensitive and as specific as expert microscopy for *P. vivax.* Gazelle™ demonstrated a limit of detection below that of the comparator rapid test. Its ease of use, short time to result, and accuracy hold potential for rapid screening for *P. vivax* in remote resource-poor locations where PCR and microscopy are not feasible and cannot immediately access to modern medical attention, but further development is required to differentiate species where necessary to guide treatment. In addition, its speed, storage capacity, rechargeable battery, cost-efficiency, and alternative to lack of microscopists makes it very practical for use in field settings. Even so, more studies are required to determine the device’s performance against different malaria species (and co-infections), using peripheral blood samples and in the field where energy and cold chains are not available.

## Data Availability

Datasets from the current study are available upon reasonable request to the corresponding author.

## Abbreviations

AUC: Area under curve
DNA: Deoxyribonucleic acid
FAP: Full analysis population
FMT-HVD: Fundação de Medicina Tropical Heitor Vieira Dourado
GZL: Gazelle™
LED: Light emitting diode
LR: Likelihood ratios
LOD: Limit of detection
LAMP: Loop mediated isothermal amplification
MOD: Magnetic-optical detection
NATs: Nucleic acid tests
PPP: Per protocol population
PVP: *Plasmodium vivax* population
POC: Point of care
PCR: Polymerase chain reaction
PPV: Positive predictive value
RDT: Rapid diagnostic test
ROC: Receiver-operating characteristics
STARD: Standards for reporting of diagnostic accuracy studies
TBS: Thick blood smear
